# 2′-Eth­oxy-1,3,3-trimethyl­spiro­[indoline-2,3′-3*H*-naphtho­[2,1-*b*][1,4]oxazine]

**DOI:** 10.1107/S1600536810022890

**Published:** 2010-06-18

**Authors:** Jian Lin, Wenxiang Chai, Yunyun Yang, Jiaojiao He, Kangying Shu

**Affiliations:** a College of Materials Science and Engineering, China Jiliang University, Hangzhou 310018, People’s Republic of China

## Abstract

In the title compound, C_24_H_24_N_2_O_2_, the five-membered ring of the indoline ring system adopts an envelope conformation with the spiro C atom at the flap. The dihedral angle between the benzene ring of the indoline ring system and the naphthalene ring system is 71.70 (7)°. In the crystal structure, pair of weak C—H⋯O hydrogen bonds link the mol­ecules into centrosymmetric dimers.

## Related literature

For applications of spiro­oxazines, see: Chibisov & Gardner (1999 ▶); Khairutdinov *et al.* (1998 ▶); Pozzo *et al.* (1993 ▶); Tan *et al.* (2005 ▶); Zhang *et al.* (2008 ▶). For related structures, see: Lin *et al.* (2009 ▶); Uznanski *et al.* (2001 ▶).
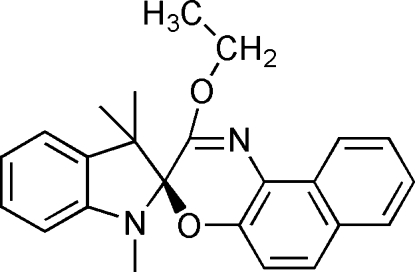


## Experimental

### 

#### Crystal data

C_24_H_24_N_2_O_2_
*M* *_r_* = 372.45
Monoclinic, 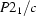

*a* = 8.6105 (4) Å
*b* = 22.9239 (8) Å
*c* = 10.2022 (5) Å
β = 93.516 (4)°
*V* = 2009.98 (15) Å^3^
*Z* = 4
Mo *K*α radiation
μ = 0.08 mm^−1^
*T* = 293 K
0.34 × 0.30 × 0.20 mm

#### Data collection

Oxford Xcalibur Gemini ultra diffractometer
Absorption correction: multi-scan (*CrysAlis PRO RED*; Oxford Diffraction, 2009 ▶) *T*_min_ = 0.974, *T*_max_ = 0.984
12650 measured reflections
4449 independent reflections
2198 reflections with *I* > 2σ(*I*)
*R*_int_ = 0.033

#### Refinement

*R*[*F* ^2^ > 2σ(*F* ^2^)] = 0.043
*wR*(*F* ^2^) = 0.085
*S* = 0.98
4449 reflections
257 parameters
H-atom parameters constrained
Δρ_max_ = 0.16 e Å^−3^
Δρ_min_ = −0.18 e Å^−3^

### 

Data collection: *CrysAlis PRO CCD* (Oxford Diffraction, 2009 ▶); cell refinement: *CrysAlis PRO CCD*; data reduction: *CrysAlis PRO RED* (Oxford Diffraction, 2009 ▶); program(s) used to solve structure: *SHELXS97* (Sheldrick, 2008 ▶); program(s) used to refine structure: *SHELXL97* (Sheldrick, 2008 ▶); molecular graphics: *SHELXTL* (Sheldrick, 2008 ▶); software used to prepare material for publication: *SHELXTL*.

## Supplementary Material

Crystal structure: contains datablocks I, global. DOI: 10.1107/S1600536810022890/is2559sup1.cif

Structure factors: contains datablocks I. DOI: 10.1107/S1600536810022890/is2559Isup2.hkl

Additional supplementary materials:  crystallographic information; 3D view; checkCIF report

## Figures and Tables

**Table 1:** Hydrogen-bond geometry (Å, °)

*D*—H⋯*A*	*D*—H	H⋯*A*	*D*⋯*A*	*D*—H⋯*A*
C18—H18⋯O1^i^	0.93	2.60	3.5014 (18)	164

Symmetry code: (i) 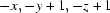.

**Fig. 1.**
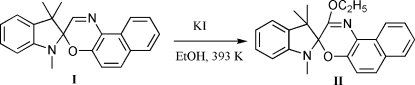
Synthesis of the title compound, (II).

**Fig. 2.**
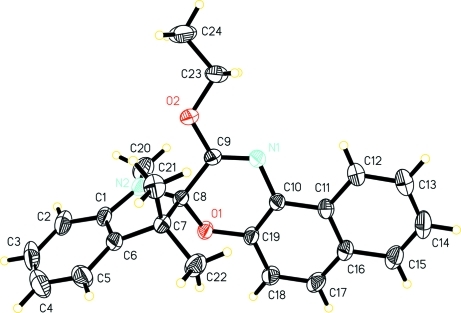
The structure of (II), showing the atom-labeling scheme of the asymmetric unit. Displacement ellipsoids are drawn at the 30% probability level and H atoms are shown as small spheres of arbitrary radii.

**Fig. 3.**
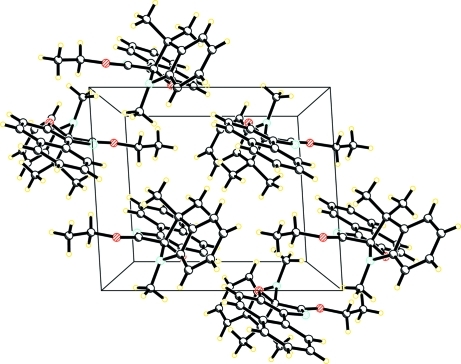
A packing diagram of (II), viewed along the a axis.

## supplementary crystallographic information

### Comment

Serve as an organic photochromic, spirooxazines have real or potential applications in many field, such as protection, decoration, display, memory, switches, photography, photometry and photomechanics (Chibisov & Gardner, 1999). For further approach to real applications, numerous types of spirooxazine derivatives have been reported over the past several decades (Pozzo *et al.*, 1993; Khairutdinov *et al.*, 1998; Zhang *et al.*, 2008). Traditionally, synthesis of spirooxazines is based on a thermal condensation reaction of the corresponding alkylidene heterocycle or its conjugate acid with *ortho*-hydroxynitroso aromatic derivatives in most polar organic solvents. To be notice, alkylidene heterocycle, such as: 1,3,3-trimethyl-2- methyleneindoline derivatives, were not stable in the air at room temperature, so they must be purified by vacuum distillation before use (Tan *et al.*, 2005). This brings to a big problem that we have to re-synthesis alkylidene heterocycle part if we want to get a novel spirooxazine with different substituents at alkylidene heterocycle moiety. According to our previous work (Lin *et al.*, 2009) in which we reported a new strategy to get a 2'-position substituted spirooxazine, a new derivative, 1,3,3-trimethyl-2'-ethoxy-1,3-dihydrospiro(indole-2,3'-naphtho(2,1-b) (1,4)oxazine), (II), has been synthesized and its crystal structure is reported here.

Since we synthesized an unexpected new organic photochromic compound, (2*S*)-2'-ethoxy-1,3,3-trimethyl-6'-(piperidin-1-yl) spiro[indoline-2,3'-3'H-naphtho[2,1-*b*][1,4]oxazine], we studied on the possibility of the ethoxy reaction at the 2'-position of spirooxazine using another spirooxazine derivative, C_22_H_20_N_2_O, (I). The title compound, C_24_H_24_N_2_O_2_, (II), was synthesized successfully (Fig. 1), which testified it is a general method to fabricate ethoxy-substituted (may be alkoxy-substituted) spirooxazines at the C2'carbon atom of the C2'=N1' bond.

The title compound, C_24_H_24_N_2_O_2_, consists of an ethoxy group bonded to parent molecule (I) at the 2'-position crystallizing with a molecule in the asymmetric unit (Fig. 2). The five-membered ring C1/N2/C6–C8 adopts an envelope conformation with the flap at C8. The dihedral angle between the benzene ring (atoms C1–C6) and the naphthalene ring (atoms C10–C19) is 71.70 (7)°. For the other 2'-position substituted derivatives (C_23_H_22_N_2_O_2_, C_27_H_24_N_2_O_2_ and C_29_H_33_N_3_O_2_), the corresponding dihedral angles are 74.2 (1), 76.5 (6) and 71.6 (2)/72.7 (2)°, respectively (Uznanski *et al.*, 2001; Lin *et al.*, 2009). The bond lengths and angles around the spiro carbon in (II) are similar to those in the other photochromic spirooxazines.

### Experimental

Potassium iodide (17 mg, 0.1 mmol) and the parent spirooxazine, 1,3,3-trimethylspiro[indoline-2,3'-[3H]-naphth[2,1-*b*][1,4]oxazine] (33 mg, 0.1 mmol) were heated in a Teflon-lined stainless steel autoclave with ethanol (10 ml) at 393 K. Block-shaped colorless crystals of (II), were obtained by slow evaporation from the filtrated reaction solution at room temperature. Yield: 45% (with reaction solution left). m.p.: 418–419 K. IR (KBr, cm^-1^): 3053, 2982, 2964, 2888, 1724, 1714, 1637, 1609, 1577, 1508, 1490, 1466, 1398, 1383, 1367, 1321, 1296, 1267, 1252, 1232, 1198, 1188, 1153, 1140, 1101, 1086, 1037, 1024, 993, 977, 952, 895, 862, 816, 781, 750, 743, 683, 655, 632, 606, 566, 551, 516, 499, 437. ^1^H NMR (CDCl_3_): δ 6.56–8.47 (10*H*, ArH), 4.58 (2*H*, CH_2_), 2.94 (3*H*, CH_3_), 1.42 (3*H*, CH_3_), 1.22 (6*H*, CH_3_). Analysis found (calculated) for C_24_H_24_N_2_O_2_: C 77.35 (77.39), H 6.60 (6.49), N 7.50% (7.52%).

### Refinement

The H atoms were placed in their calculated positions (C—H = 0.93–0.97 Å) and included in the refinement using the riding model, with *U*_iso_(H) = 1.2 or 1.5*U*_eq_(C).

### Crystal data

**Table:**

C_24_H_24_N_2_O_2_	*F*(000) = 792
*M**_r_* = 372.45	*D*_x_ = 1.231 Mg m^−^^3^
Monoclinic, *P*2_1_/*c*	Melting point: 418 K
Hall symbol: -P 2ybc	Mo *K*α radiation, λ = 0.71073 Å
*a* = 8.6105 (4) Å	Cell parameters from 3384 reflections
*b* = 22.9239 (8) Å	θ = 3.2–27.3°
*c* = 10.2022 (5) Å	µ = 0.08 mm^−^^1^
β = 93.516 (4)°	*T* = 293 K
*V* = 2009.98 (15) Å^3^	Block, colorless
*Z* = 4	0.34 × 0.30 × 0.20 mm

### Data collection

**Table:**

Oxford Xcalibur Gemini ultra diffractometer	4449 independent reflections
Radiation source: fine-focus sealed tube	2198 reflections with *I* > 2σ(*I*)
graphite	*R*_int_ = 0.033
Detector resolution: 10.3592 pixels mm^-1^	θ_max_ = 27.3°, θ_min_ = 3.3°
ω scans	*h* = −11→10
Absorption correction: multi-scan (*CrysAlis PRO RED*; Oxford Diffraction, 2009)	*k* = −29→28
*T*_min_ = 0.974, *T*_max_ = 0.984	*l* = −13→13
12650 measured reflections	

### Refinement

**Table:**

Refinement on *F*^2^	Primary atom site location: structure-invariant direct methods
Least-squares matrix: full	Secondary atom site location: difference Fourier map
*R*[*F*^2^ > 2σ(*F*^2^)] = 0.043	Hydrogen site location: inferred from neighbouring sites
*wR*(*F*^2^) = 0.085	H-atom parameters constrained
*S* = 0.98	*w* = 1/[σ^2^(*F*_o_^2^) + (0.030*P*)^2^] where *P* = (*F*_o_^2^ + 2*F*_c_^2^)/3
4449 reflections	(Δ/σ)_max_ < 0.001
257 parameters	Δρ_max_ = 0.16 e Å^−^^3^
0 restraints	Δρ_min_ = −0.18 e Å^−^^3^

### Special details

**Table:**

Geometry. All e.s.d.'s (except the e.s.d. in the dihedral angle between two l.s. planes) are estimated using the full covariance matrix. The cell e.s.d.'s are taken into account individually in the estimation of e.s.d.'s in distances, angles and torsion angles; correlations between e.s.d.'s in cell parameters are only used when they are defined by crystal symmetry. An approximate (isotropic) treatment of cell e.s.d.'s is used for estimating e.s.d.'s involving l.s. planes.
Refinement. Refinement of *F*^2^ against ALL reflections. The weighted *R*-factor *wR* and goodness of fit *S* are based on *F*^2^, conventional *R*-factors *R* are based on *F*, with *F* set to zero for negative *F*^2^. The threshold expression of *F*^2^ > σ(*F*^2^) is used only for calculating *R*-factors(gt) *etc*. and is not relevant to the choice of reflections for refinement. *R*-factors based on *F*^2^ are statistically about twice as large as those based on *F*, and *R*- factors based on ALL data will be even larger.

### Fractional atomic coordinates and isotropic or equivalent isotropic displacement parameters (Å^2^)

**Table:**

	*x*	*y*	*z*	*U*_iso_*/*U*_eq_	
O1	0.10563 (10)	0.53152 (4)	0.31415 (9)	0.0480 (3)	
O2	0.22203 (12)	0.57809 (4)	0.00532 (9)	0.0593 (3)	
N1	0.23974 (13)	0.48659 (5)	0.09366 (11)	0.0489 (3)	
N2	0.09544 (13)	0.62319 (5)	0.21564 (12)	0.0482 (3)	
C1	0.13539 (18)	0.66314 (6)	0.31558 (15)	0.0487 (4)	
C2	0.0555 (2)	0.71249 (7)	0.3510 (2)	0.0713 (5)	
H2	−0.0378	0.7234	0.3069	0.086*	
C3	0.1210 (3)	0.74527 (7)	0.4557 (2)	0.0930 (7)	
H3	0.0704	0.7789	0.4814	0.112*	
C4	0.2579 (3)	0.72923 (9)	0.5217 (2)	0.0945 (7)	
H4	0.2972	0.7511	0.5929	0.113*	
C5	0.3369 (2)	0.68093 (7)	0.48276 (17)	0.0704 (5)	
H5	0.4308	0.6704	0.5263	0.085*	
C6	0.27635 (18)	0.64807 (6)	0.37867 (14)	0.0473 (4)	
C7	0.34049 (16)	0.59543 (6)	0.31284 (14)	0.0450 (4)	
C8	0.19045 (15)	0.57223 (5)	0.23417 (13)	0.0410 (3)	
C9	0.21867 (16)	0.54118 (6)	0.10658 (13)	0.0454 (4)	
C10	0.22059 (15)	0.45194 (5)	0.20565 (14)	0.0416 (3)	
C11	0.26861 (15)	0.39259 (6)	0.20617 (15)	0.0441 (4)	
C12	0.34693 (17)	0.36818 (6)	0.10200 (16)	0.0568 (4)	
H12	0.3668	0.3910	0.0296	0.068*	
C13	0.39369 (19)	0.31155 (7)	0.1063 (2)	0.0715 (5)	
H13	0.4463	0.2961	0.0372	0.086*	
C14	0.3635 (2)	0.27640 (7)	0.2132 (2)	0.0758 (6)	
H14	0.3962	0.2377	0.2150	0.091*	
C15	0.2869 (2)	0.29814 (6)	0.31467 (19)	0.0671 (5)	
H15	0.2666	0.2741	0.3850	0.081*	
C16	0.23744 (16)	0.35707 (6)	0.31460 (16)	0.0498 (4)	
C17	0.16004 (19)	0.38141 (6)	0.41846 (16)	0.0620 (5)	
H17	0.1372	0.3580	0.4891	0.074*	
C18	0.11748 (17)	0.43869 (6)	0.41815 (15)	0.0575 (4)	
H18	0.0670	0.4542	0.4883	0.069*	
C19	0.15037 (16)	0.47384 (5)	0.31146 (14)	0.0438 (4)	
C20	−0.06197 (19)	0.61855 (7)	0.15824 (19)	0.0748 (5)	
H20A	−0.1290	0.6046	0.2232	0.112*	
H20B	−0.0643	0.5918	0.0857	0.112*	
H20C	−0.0971	0.6562	0.1277	0.112*	
C21	0.46191 (17)	0.61472 (6)	0.21791 (16)	0.0620 (5)	
H21A	0.4164	0.6429	0.1573	0.093*	
H21B	0.4962	0.5815	0.1703	0.093*	
H21C	0.5492	0.6319	0.2667	0.093*	
C22	0.4158 (2)	0.55129 (6)	0.40929 (17)	0.0717 (5)	
H22A	0.5025	0.5691	0.4574	0.108*	
H22B	0.4513	0.5182	0.3618	0.108*	
H22C	0.3408	0.5388	0.4693	0.108*	
C23	0.2486 (2)	0.55342 (7)	−0.12201 (15)	0.0756 (5)	
H23A	0.1723	0.5234	−0.1445	0.091*	
H23B	0.3516	0.5362	−0.1213	0.091*	
C24	0.2344 (2)	0.60208 (8)	−0.21924 (17)	0.0930 (6)	
H24A	0.1314	0.6182	−0.2204	0.139*	
H24B	0.2537	0.5875	−0.3050	0.139*	
H24C	0.3090	0.6319	−0.1949	0.139*	

### Atomic displacement parameters (Å^2^)

**Table:**

	*U*^11^	*U*^22^	*U*^33^	*U*^12^	*U*^13^	*U*^23^
O1	0.0591 (6)	0.0358 (5)	0.0510 (6)	0.0043 (5)	0.0201 (5)	0.0047 (4)
O2	0.0940 (8)	0.0462 (6)	0.0382 (6)	−0.0067 (5)	0.0094 (5)	0.0066 (5)
N1	0.0654 (8)	0.0389 (7)	0.0436 (8)	−0.0023 (6)	0.0126 (6)	−0.0006 (6)
N2	0.0505 (8)	0.0390 (7)	0.0543 (8)	0.0086 (6)	−0.0031 (6)	0.0064 (6)
C1	0.0630 (10)	0.0305 (8)	0.0545 (10)	0.0000 (7)	0.0183 (8)	0.0056 (7)
C2	0.0823 (12)	0.0414 (10)	0.0938 (15)	0.0106 (9)	0.0344 (11)	0.0083 (10)
C3	0.1261 (19)	0.0404 (10)	0.1195 (19)	−0.0058 (12)	0.0634 (16)	−0.0214 (12)
C4	0.1252 (19)	0.0703 (14)	0.0921 (17)	−0.0293 (13)	0.0388 (15)	−0.0338 (12)
C5	0.0889 (13)	0.0617 (11)	0.0616 (12)	−0.0206 (10)	0.0123 (10)	−0.0123 (9)
C6	0.0604 (10)	0.0366 (8)	0.0458 (9)	−0.0056 (7)	0.0099 (8)	0.0008 (7)
C7	0.0509 (9)	0.0378 (8)	0.0460 (9)	0.0027 (7)	0.0004 (7)	0.0055 (7)
C8	0.0486 (9)	0.0349 (7)	0.0401 (9)	0.0017 (7)	0.0076 (7)	0.0061 (6)
C9	0.0567 (10)	0.0419 (9)	0.0381 (9)	−0.0045 (7)	0.0063 (7)	0.0043 (7)
C10	0.0487 (9)	0.0344 (8)	0.0426 (9)	−0.0033 (6)	0.0088 (7)	0.0003 (7)
C11	0.0401 (8)	0.0364 (8)	0.0557 (10)	−0.0027 (7)	0.0026 (7)	−0.0040 (7)
C12	0.0566 (10)	0.0454 (9)	0.0688 (12)	0.0018 (8)	0.0064 (9)	−0.0114 (8)
C13	0.0694 (12)	0.0547 (11)	0.0904 (15)	0.0075 (9)	0.0040 (10)	−0.0217 (10)
C14	0.0750 (13)	0.0415 (10)	0.1086 (18)	0.0119 (9)	−0.0133 (12)	−0.0127 (11)
C15	0.0749 (12)	0.0407 (9)	0.0841 (14)	−0.0019 (8)	−0.0092 (11)	0.0060 (9)
C16	0.0506 (10)	0.0356 (8)	0.0629 (11)	−0.0028 (7)	0.0004 (8)	0.0021 (8)
C17	0.0761 (12)	0.0466 (10)	0.0646 (12)	−0.0051 (8)	0.0152 (9)	0.0186 (8)
C18	0.0722 (11)	0.0476 (9)	0.0555 (11)	0.0025 (8)	0.0262 (8)	0.0082 (8)
C19	0.0493 (9)	0.0328 (8)	0.0503 (10)	0.0007 (7)	0.0117 (7)	0.0039 (7)
C20	0.0609 (12)	0.0686 (11)	0.0928 (14)	0.0078 (9)	−0.0135 (10)	0.0146 (10)
C21	0.0533 (10)	0.0603 (10)	0.0734 (12)	−0.0029 (8)	0.0120 (9)	−0.0050 (8)
C22	0.0805 (12)	0.0560 (10)	0.0748 (13)	0.0045 (9)	−0.0259 (10)	0.0107 (9)
C23	0.1160 (15)	0.0711 (11)	0.0406 (11)	−0.0154 (10)	0.0133 (10)	−0.0001 (9)
C24	0.1305 (17)	0.1011 (15)	0.0468 (12)	−0.0337 (13)	0.0011 (11)	0.0163 (10)

### Geometric parameters (Å, °)

**Table:**

O1—C19	1.3780 (14)	C12—C13	1.3591 (19)
O1—C8	1.4640 (15)	C12—H12	0.9300
O2—C9	1.3371 (15)	C13—C14	1.393 (2)
O2—C23	1.4478 (18)	C13—H13	0.9300
N1—C9	1.2724 (16)	C14—C15	1.356 (2)
N1—C10	1.4095 (16)	C14—H14	0.9300
N2—C1	1.3977 (18)	C15—C16	1.417 (2)
N2—C8	1.4320 (16)	C15—H15	0.9300
N2—C20	1.4471 (18)	C16—C17	1.402 (2)
C1—C6	1.3823 (19)	C17—C18	1.3631 (19)
C1—C2	1.383 (2)	C17—H17	0.9300
C2—C3	1.397 (3)	C18—C19	1.3971 (19)
C2—H2	0.9300	C18—H18	0.9300
C3—C4	1.372 (3)	C20—H20A	0.9600
C3—H3	0.9300	C20—H20B	0.9600
C4—C5	1.371 (3)	C20—H20C	0.9600
C4—H4	0.9300	C21—H21A	0.9600
C5—C6	1.378 (2)	C21—H21B	0.9600
C5—H5	0.9300	C21—H21C	0.9600
C6—C7	1.5025 (19)	C22—H22A	0.9600
C7—C22	1.5278 (18)	C22—H22B	0.9600
C7—C21	1.5332 (19)	C22—H22C	0.9600
C7—C8	1.5713 (18)	C23—C24	1.493 (2)
C8—C9	1.5163 (18)	C23—H23A	0.9700
C10—C19	1.3649 (19)	C23—H23B	0.9700
C10—C11	1.4218 (17)	C24—H24A	0.9600
C11—C12	1.409 (2)	C24—H24B	0.9600
C11—C16	1.4125 (19)	C24—H24C	0.9600
			
C19—O1—C8	116.81 (10)	C14—C13—H13	119.7
C9—O2—C23	117.27 (11)	C15—C14—C13	120.53 (16)
C9—N1—C10	116.47 (12)	C15—C14—H14	119.7
C1—N2—C8	108.97 (11)	C13—C14—H14	119.7
C1—N2—C20	121.73 (13)	C14—C15—C16	120.68 (17)
C8—N2—C20	120.44 (11)	C14—C15—H15	119.7
C6—C1—C2	121.30 (15)	C16—C15—H15	119.7
C6—C1—N2	110.22 (12)	C17—C16—C11	118.98 (13)
C2—C1—N2	128.46 (15)	C17—C16—C15	122.42 (15)
C1—C2—C3	117.11 (17)	C11—C16—C15	118.60 (15)
C1—C2—H2	121.4	C18—C17—C16	121.46 (14)
C3—C2—H2	121.4	C18—C17—H17	119.3
C4—C3—C2	121.72 (18)	C16—C17—H17	119.3
C4—C3—H3	119.1	C17—C18—C19	119.34 (14)
C2—C3—H3	119.1	C17—C18—H18	120.3
C5—C4—C3	120.08 (19)	C19—C18—H18	120.3
C5—C4—H4	120.0	C10—C19—O1	120.43 (12)
C3—C4—H4	120.0	C10—C19—C18	121.66 (12)
C4—C5—C6	119.59 (18)	O1—C19—C18	117.88 (13)
C4—C5—H5	120.2	N2—C20—H20A	109.5
C6—C5—H5	120.2	N2—C20—H20B	109.5
C5—C6—C1	120.14 (15)	H20A—C20—H20B	109.5
C5—C6—C7	130.64 (15)	N2—C20—H20C	109.5
C1—C6—C7	109.21 (12)	H20A—C20—H20C	109.5
C6—C7—C22	113.40 (13)	H20B—C20—H20C	109.5
C6—C7—C21	109.53 (11)	C7—C21—H21A	109.5
C22—C7—C21	108.61 (12)	C7—C21—H21B	109.5
C6—C7—C8	100.77 (11)	H21A—C21—H21B	109.5
C22—C7—C8	114.07 (11)	C7—C21—H21C	109.5
C21—C7—C8	110.25 (12)	H21A—C21—H21C	109.5
N2—C8—O1	107.05 (10)	H21B—C21—H21C	109.5
N2—C8—C9	112.96 (11)	C7—C22—H22A	109.5
O1—C8—C9	106.91 (10)	C7—C22—H22B	109.5
N2—C8—C7	103.68 (10)	H22A—C22—H22B	109.5
O1—C8—C7	110.71 (10)	C7—C22—H22C	109.5
C9—C8—C7	115.30 (11)	H22A—C22—H22C	109.5
N1—C9—O2	122.16 (13)	H22B—C22—H22C	109.5
N1—C9—C8	125.60 (12)	O2—C23—C24	107.05 (14)
O2—C9—C8	112.23 (11)	O2—C23—H23A	110.3
C19—C10—N1	120.89 (12)	C24—C23—H23A	110.3
C19—C10—C11	119.45 (13)	O2—C23—H23B	110.3
N1—C10—C11	119.60 (13)	C24—C23—H23B	110.3
C12—C11—C16	118.97 (13)	H23A—C23—H23B	108.6
C12—C11—C10	121.99 (14)	C23—C24—H24A	109.5
C16—C11—C10	119.04 (13)	C23—C24—H24B	109.5
C13—C12—C11	120.65 (16)	H24A—C24—H24B	109.5
C13—C12—H12	119.7	C23—C24—H24C	109.5
C11—C12—H12	119.7	H24A—C24—H24C	109.5
C12—C13—C14	120.55 (17)	H24B—C24—H24C	109.5
C12—C13—H13	119.7		

### Hydrogen-bond geometry (Å, °)

**Table:**

*D*—H···*A*	*D*—H	H···*A*	*D*···*A*	*D*—H···*A*
C18—H18···O1^i^	0.93	2.60	3.5014 (18)	164

Symmetry codes: (i) −*x*, −*y*+1, −*z*+1.
